# Health care professionals’ experiences of supporting persons with metabolic risk factors to increase their physical activity level – a qualitative study in primary care

**DOI:** 10.1080/02813432.2023.2187668

**Published:** 2023-03-16

**Authors:** Kristina Larsson, Maria Hagströmer, Jenny Rossen, Unn-Britt Johansson, Åsa Norman

**Affiliations:** a Department of Health Promoting Science, Sophiahemmet University, Stockholm, Sweden; b Division of Physiotherapy, Department of Neurobiology, Care Sciences and Society, Karolinska Institutet, Stockholm, Sweden; c Academic Primary Care Centre, Region Stockholm, Stockholm, Sweden; d Department of Clinical Science and Education, Södersjukhuset, Karolinska Institutet, Stockholm, Sweden; e Department of Clinical Neuroscience, Karolinska Institutet, Stockholm, Sweden; f Department of Psychology, Stockholm University, Stockholm, Sweden

**Keywords:** Barriers, facilitators, health care professionals, physical activity, primary care, Sweden

## Abstract

**Objective:**

To be regularly physically active is of major importance for the health of people with metabolic risk factors. Many of these persons are insufficiently active and in need of support. This study aimed to explore barriers and facilitators perceived by health care professionals’ within Swedish primary care in their work to support persons with metabolic risk factors to increase their physical activity.

**Design:**

A qualitative design with focus group discussions was used. The data were analysed using qualitative content analysis with a manifest, inductive approach.

**Setting:**

Primary health care in five Swedish healthcare regions.

**Subjects:**

Nine physiotherapists, ten physicians and five nurses participated in six digital focus group discussions including two to six participants.

**Results:**

Barriers and facilitators to supporting persons with metabolic risk factors to increase their physical activity were found within four generic categories, where the barriers and facilitators related to each generic category: ‘Patient readiness for change’, ‘Supporting the process of change’, ‘The professional role’, and ‘The organisation of primary care’.

**Conclusion:**

The findings suggests that barriers and facilitators for supporting patients with metabolic risk factors can be found at several levels within primary care, from individual patient and the health care professionals to the organisational level. In the primary care setting, this should be highlighted when implementing support to increase physical activity in people with metabolic risk factors.

**KEY POINTS**
- Health care professionals within primary care are in a position to support people with metabolic risk factors to increase their physical activity.
- Barriers and facilitators to support the patients should be addressed at several levels within primary care.
- The study highlights factors on multiple levels such as professional responsibility, organisational prioritisation and resources, and the challenge to motivate behaviour change.

## Introduction

There is high quality evidence that regular physical activity and limited time spent sedentary can prevent non-communicable diseases such as cardiovascular disease, type 2 diabetes, and cancer [1–3]. However, about one-third of the adult population globally is not physically active enough to confer health benefits, and the numbers are higher in high-income countries compared to low- and middle-income countries [4]. Also, physical inactivity is more common in people with chronic conditions [5]. It is recommended that all adults should engage in physical activity of at least moderate intensity for 150–300 min per week, perform strength training at least twice a week, and limit sedentary time [6]. A dose–response relationship exists between physical activity and health, with the most significant potential benefits being for sedentary individuals to start with some activity, even if the recommended dose is not attained [2,7].

Around 20–25% of adults globally are diagnosed with metabolic syndrome, defined as having central obesity plus any two of the following risk factors: raised levels of triglycerides, reduced HDL cholesterol levels, raised blood pressure, and raised fasting plasma glucose levels [8,9]. To be physically active on a regular basis is of major importance for people with metabolic risk factors, since each of the above-mentioned risk factors is individually favourably influenced by physical activity [10].

To reduce physical inactivity levels globally, the World Health Organization recommends that all countries should integrate physical activity counselling into primary care [11]; this is also highlighted in Swedish national guidelines for professional caregivers [12]. Moreover, in Sweden, a national programme to counteract unhealthy lifestyle habits has been developed, including explicit strategies for how to perform support for the prevention and treatment of low physical activity levels [13]. The recommended measures are brief advice with additionally recommending an activity tracker and/or physical activity on prescription (PAP) or a web-based intervention, or counselling (e.g. based on the person-centred conversational style motivational interviewing) [13,14]. PAP consists of three parts: a person-centred dialogue; a written prescription with individually tailored physical activity recommendations; and follow-up consultation [15]. Moreover, in Swedish primary care, counselling and lifestyle modification can have positive effect on cardiovascular risk factors [16,17]. However, there are large differences between the regions in Sweden regarding support to patients to increase their physical activity [18], indicating a need for further implementation.

Since health care professionals come in contact with a large proportion of the population, they play a key role in the preventive work with physical activity [14]. Within the Swedish primary care, some of the most common professions are physicians, nurses, physiotherapists, but other professions can exist, such as health educators. The professions can support patients in becoming more physically active, and five or more contacts as well as longer follow-ups are associated with greater improvements [19]. A systematic review has shown that health care professionals considered physical activity promotion as part of their professional role and of importance for their patients [20], and many patients want support to change their lifestyle habits [21].

It is known that barriers for the implementation of work procedures or innovations in organisations may arise at multiple levels. The Consolidated Framework For Implementation Research (CFIR) is a compilation of a number of intervention theories and frameworks and provides guidance regarding factors of importance to consider for successful implementation of an intervention. The CFIR describes that five domains representing different levels within and around the health care organisation, are of importance to consider when trying to implement an intervention in the health care setting. These five domains are intervention characteristics, outer setting (social, political and economic context surrounding the organisation), inner setting (the organisation in which the implementation process will occur), characteristics of the individuals involved (e.g. health care professionals), and the process of implementation [22].

To better understand how professions within primary care can support people with metabolic risk factors to increase their physical activity, and identify ways to improve the implementation of the support work within the primary care, more knowledge is needed about health care professionals’ experiences of barriers and facilitators to supporting these people. Therefore, the aim of this study was to explore barriers and facilitators perceived by health care professionals within Swedish primary care in their work to support persons with metabolic risk factors to increase their physical activity.

## Materials and methods

### Study design

A qualitative design was used to explore nurses’, physicians’, and physiotherapists’ experiences of supporting patients with metabolic risk factors to physical activity. This design is suitable when studying peoples’ lived experiences [23]. The study is reported according to the COREQ 32-item checklist for qualitative studies [24], which can be found in Appendix A. The data were collected by digital focus group discussions. In focus group methodology, data are generated through interaction between participants, encouraging participants to clarify and explore their opinions. Focus groups are useful when describing experiences and perceptions of an area that the participants have in common [25].

### Setting and participants

The study was carried out within the Swedish primary care, where the most common professions are physicians, nurses, physiotherapists, dieticians, occupational therapists, psychologists, midwives, and speech therapist. However, other professions, such as health educators can exist, even though it is unusual.

Convenience sampling was used to identify health care professionals (nurses, physicians, and physiotherapists) working clinically in the primary care setting and meeting patients with metabolic risk factors. All employed nurses, physicians, and physiotherapists at 27 primary care centres or rehab centres in five regions of Sweden were invited by email after approval from their unit managers. In total, 282 were invited to participate, 78 declined, 164 did not respond and 40 agreed to participate. Out of the 40 who agreed to participate, 16 could not find a suitable time. A final sample of 24 were included, consisting of 9 physiotherapists, 10 physicians and 5 nurses. Details of the group characteristics are described in Table 1.

**Table 1.** Number of focus groups, profession, number of participants and years of clinical experience.

Focus group ID	Profession	Number of participants (men/women)	Number of regions represented in each group	Experience from clinical work in primary care	
				≤5 years, *n*	>5 years, *n*
FGPT1	Physiotherapists	3 (0/3)	2	2	1
FGPT2	Physiotherapists	6 (1/5)	3	4	2
FGPH1	Physicians	6 (0/6)	2	3	3
FGPH2	Physicians	4 (1/3)	2	3	1
FGNU1	Nurses	3 (0/3)	2	1	2
FGNU2	Nurses	2 (0/2)	2	1	1

FGPT: Focus group with physiotherapists; FGPH: Focus group with physicians; FGNU: Focus group with nurses.

### Data collection

Six digital focus group discussions were conducted, two with each profession with two to six participants in each group, from June to November 2021. A semi-structured topic guide with open-ended questions was developed based on the question categories described by Krueger and Casey [25], including introductory questions, transition questions, key questions and ending questions. The questions were inspired by the domains outer setting, inner setting and characteristics of the individuals involved, from the implementation framework Consolidated Framework for Implementation Research (CFIR), which also highlights the importance of addressing possible barriers and facilitators at multiple levels in and around health care organisations [22]. CFIR was used to make sure that no important aspect from previous implementation research was missing in the topic guide. In addition, the questions were formulated to relate to areas highlighted by the National Board of Health and Welfare, for preventive work within health care [26]. Probing questions were used to get a deeper understanding of the participants’ answers and to facilitate interaction between participants, e.g. ‘Can you tell me a bit more about that?’ The topic areas for the questions were; Description of the meeting with the patient, Health care professionals’ views of the patients’ need for physical activity support, Health care professionals’ own knowledge and competence and The workplace and the organization. The full topic guide can be found in Appendix B.

The topic guide was pilot tested with a nurse specialised in diabetes, resulting in some minor adjustments. Prior to the focus group discussions, the participants received information about the study, answered a questionnaire including demographic questions and gave written informed consent. All discussions started with general information including the aim of the study, the moderator’s profession and background, information about how the data from the discussions would be stored, encouraging the participants to tell all their experiences, both positive and negative, the possibility to withdraw from the study at any time, as well as letting the participants introduce themselves. The moderator did not have any prior relationship with the participants. The focus group discussions were performed via a digital meeting platform and recorded with an external audio recorder. A moderator (KL) led the discussion, and an assistant (ÅN or YW) took notes if something deviated from the pre-planned procedure and helped with technical issues during the discussions. KL (female) is a health educator and is currently a doctoral student, and had no previous experience in conducting focus group discussions. ÅN (female) is an Associate Professor and an anthropologist with extensive experience in behavioural science as well as qualitative research, who has not worked within primary care. YW (male) is a registered nurse and doctoral student. The focus group discussions had a duration between 60 and 90 min, including introduction of the discussions. The participants did not receive any economic compensation for their participation.

### Ethical consideration

The Swedish Ethical Review Authority in Stockholm approved the study (Dnr. 2021-00752). Prior to the focus group discussions all participants signed an informed consent document which was sent and returned by post.

### Data analysis

The data were analysed using qualitative content analysis [27] with an manifest, inductive approach [28]. KL listened to the audio recordings of each focus group discussion several times and transcribed them verbatim. Meaning units indicating barriers or facilitators to support patients to physical activity were highlighted in the text and labelled with an appropriate code describing ‘the what’ within the meaning unit (by KL). To ensure credibility, ÅN independently selected meaning units, intercompared and matched them with those selected by KL. Also, for the first two focus groups discussions, ÅN continuously checked the coding made by KL, and they discussed until consensus was reached. After the coding, higher order headings were identified by grouping together codes with similar content and meaning. Thereafter, these higher order headings were described in text, and chunks of text were collapsed into sub-categories and generic categories where similarities existed. To ensure credibility, this step consisted of going back and forth to the original text, as well as a continuous discussion between KL and ÅN until consensus was reached. Ultimately, the identified higher order headings were discussed with all authors (KL, MH, JR, U-BJ and ÅN) until consensus was reached. The analysis was initially performed separately for each profession (nurses, physicians and physiotherapists), to see if any differences between the professions emerged. For sub-categories with distinct differences between the professions, the professions’ experiences were kept separate. However, when no major differences between professions were identified, data were collapsed to convey a complete view of health care professionals’ experiences. Also, barriers and facilitators were separated in the analysis process. All analyses were conducted manually; no software was used.

The focus groups were assigned abbreviations for their profession and group number, e.g. FGPT1 (see Table 1 for all groups). Also, participants within each focus group were assigned an individual number, e.g. PT2, to ensure anonymity. Explanation of the context are written within square brackets [X]. All discussions were conducted and transcribed in Swedish. After finalising the analysis, the results were translated into English by KL.

## Results

The qualitative content analysis resulted in perceived barriers and facilitators by the health care professionals in four generic categories: (1) Patient readiness for change; (2) Supporting the process of change; (3) The professional role, and; (4) The organisation of primary care. Generic categories and sub-categories are presented in Table 2. A table including barriers and facilitators is presented in Appendix C.

**Table 2.** Overview of generic categories and sub-categories identified from focus group discussions with the health care professionals.

Generic categories	Patient readiness for change	Supporting the process of change	The professional role	The organisation of primary care
Sub-categories	The patient’s attitude to physical activity	The conversation that promotes motivation and behaviour change	The health care professional’s own view	Support from management
	The patient’s insight into their own disease	Usability of tools for supporting physical activity	The views of the profession responsible for physical activity	Priorities of time and resources connected to economic consequences
			The own knowledge and competence	The role of prevention and routines within primary care
			Collaboration between professions	Primary care collaboration with other actors

### Patient readiness for change

This generic category described how the participants work to promote physical activity in patients with metabolic risk factors was influenced by the patient’s attitude to being physically active and the patient’s insight regarding their own diagnosis.

#### The patient’s attitude to physical activity

The participants experienced that the patient’s personal attitude towards physical activity varied based on self-awareness, interest, and perceived barriers to physical activity. On the one hand, the participants perceived that if the patients were knowledgeable and self-aware, wished for a higher physical activity level and actively sought help, these factors served as facilitators for supporting patients to increase their physical activity. On the other hand, if the patients seemed to lack knowledge, had low self-awareness, self-reported unrealistic activity levels, and did not seek help themselves, these factors acted as barriers. The latter patients often seemed to experience obstacles to being physically active such as pain, overweight, fear, lack of knowledge, mental illness, bad weather, or the covid-19 pandemic. The participants were surprised by the patients’ low activity levels and lack of a sense of responsibility for their own health, resulting in worries for their patients’ future.

NU1: they know that [they need to increase their activity]. But no, it’s hard getting started so they sit in their chair and, well. Eat a bit and watch TV.

NU2: [They] get even more tired…

NU1: Yeah, that’s what it is, the vicious circle actually.

NU2: Yeah… (FGNU2)

#### The patient’s insight into their own disease

Barriers and facilitators in this sub-category highlighted patients’ emotional connection to and establishment of the diagnosis.

A barrier in the supportive work was the patients’ feelings of guilt and shame for their unhealthy lifestyle, choices that may also have caused the current disease. The guilt and shame seemed to fuel hopelessness in the patients and resignation that prevented them from even trying to live healthier. The participants perceived that it was easier to motivate patients with established type 2 diabetes, as these patients experienced tangible consequences and benefits of physical activity. While patients who had not developed type 2 diabetes, but needed support to increase their physical activity, were harder to motivate, as the benefits of activity were less visible to them.

PH3: when you ask [the patients], then nobody is physically inactive […] I don’t know if they feel a bit ashamed or something like that, and say “I do actually get some exercise, a bit anyway.” I find that a really common reaction […]

PH4: […] I agree with you that many are a bit ashamed about, well "I know I ought to do more, but at least I’m doing these things and that’s always something”, but are ashamed that they’ve ended up in the situation they have maybe. (FGPH2)

### Supporting the process of change

This generic category described the conversation with the patient in which the participants was trying to improve patient motivation and promote behaviour change, as well as using different methods and practical tools to help the patient increase their physical activity level.

#### The conversation that promotes motivation and behaviour change

The participants experienced both barriers and facilitators in creating a conversation with the patient which would improve motivation and support change in the patient’s activity behaviours. They perceived that it was easier to discuss physical activity compared to other lifestyle habits. Facilitators mentioned by the participants were mainly related to success in creating a positive climate of change together with the patient. In the meeting, the participants found that talking about physical activity often and in an unconditional manner, starting with general questions, and daring to ask the patient difficult questions, were facilitators in the supportive work. In addition, the participants found that adapting the conversation to focus on the specific patient’s expectations and prerequisites, and suggesting small steps made it easier to overcome the patient’s resistance towards behaviour change. The participants tried to adjust the support to the patient’s individual needs: some wanted a lot of support, while others wanted to try to make the behaviour change by themselves. The participants also described facilitators related to preparing the patient for the topic of the meeting when booking it, using motivational interviewing, setting up goals and sub-goals together with the patient, discussing with the patient how clinical parameters improved and worsened over time, booking a follow up meeting, and focusing on reducing blood glucose levels instead of losing weight, since reducing blood glucose has a quicker effect and can therefore spur more motivation. The participants also felt that it was fun and rewarding to be part of the patient’s journey and help with the behaviour change. Although the participants endeavoured to give all patients a chance in the patient–professional meeting, they found that a lack of patient motivation made the preventive work impossible and that they struggled when patients did not show any indication of willingness to change their behaviour. Also, the participants found it hard to establish a dialogue with unmotivated patients with the result that these patients were not prioritised.

NU1: I try saying, “But if you just go out for a walk for 15 minutes,” I mean, if I say 30 they get really tired, that sounds like a long time, yeah, but 15 minutes, a short while, I usually say, eh, well yeah, but that can work, some actually do it.

NU2: It’s good to start with something small, something achievable.

NU1: Take a walk around the house, or I’ve said if it’s bad weather, a lot of them are old, 80-90-years, with high lab values and more, and hard to motivate to go out for a walk. [So I tell them] “Well, take a walk down to the basement and then walk upstairs to the third floor.” (FGNU2)

#### Usability of tools for supporting physical activity

The participants experienced that tools available to help patients increase their physical activity can act both as barriers and facilitators to behaviour change.

The participants identified several tools as facilitators to support the patient in increasing physical activity: they encouraged patients to use step counter apps in their cell phone, provided exercise programme hand-outs, advised patients to exercise in front of the TV or go to group exercises, prescribed PAP, prescribed medication as an add-on to the physical activity, and prepared the patients before the appointment by letting them respond to a questionnaire about their physical activity. Barriers in the work to support the patients included counselling over telephone when an interpreter was needed, the logistics around arranging patient groups, and use of subjective means of estimating the patient’s current physical activity level. Several barriers were related to PAP, such as lack of economic compensation for PAP; it was also hard to know whether the patient had grasped the message the PAP was intended to convey, and the PAP form itself was challenging to use, which ultimately meant fewer prescriptions to the patients.

PT6: Sometimes I think that it can help them [the patients] a bit if you prescribe a PAP, uh, I perceive that some are helped a lot by having it on a piece of paper in front of them, that this is what I’m supposed to do […]

PT4: I also think that it’s really good, eh, I also think that many like the step counter in the cell phone […] Because then you can see so clearly that, well today have I walked 1000 steps extra, then they’re a bit proud when they show that. So I usually think that’s is a good factor for motivation. (FGPT2)

### The professional role

This generic category described how the participants perceived their own professional role and the role of other professions within the primary care. This included the view on knowledge and competence, if any profession had a pronounced responsibility for supporting the patients, but also collaboration between professions.

#### The health care professional’s own view

Barriers and facilitators in this sub-category included the participants personal attitude towards their own ability to support the patients, the importance of the supportive work, and how they themselves were affected by the supportive work. The participants agreed that supporting patients to increase their physical activity was an important part of the treatment of metabolic risk factors. The perceived importance of giving support seemed to constitute a facilitator. It was also perceived as important that a healthy patient means less work for the health care system and thus for them as health care professionals. Another facilitator – which was in itself rewarding and increased the participants motivation in their work – was feeling that one was successful in giving the patients the support they needed. The same was true of managing to support the patients in challenging situations, e.g. when they perceived a lot of barriers to increasing their physical activity. One barrier the participants perceived was that supporting patients with metabolic risk factors to increase their physical activity was difficult, since the patients’ disease had progressed over a long time period of unhealthy lifestyle habits, making it harder to achieve a change in behaviour.

PT3: That challenge, I think it’s one of the most fun parts, to, uh, be able to push a bit and uh, uh be there for the patients so maybe they’ll succeed with those small steps. […]

PT1: In this behaviour change it’s a super important time to intervene

PT3: Yeah […]

PT1: […] to follow the patient during this change process and to see it happen and confirm that you see it and measure it and be able to give feedback to the patient, that is the reward. (FGPT2)

#### The views of the profession responsible for physical activity

Professional responsibility and how this was handled reflected both barriers and facilitators to efforts to support the patients to physical activity. As a facilitator, all participants (nurses, physicians and physiotherapists) perceived that their own profession was responsible for supporting patients with metabolic risk factors to increase their physical activity, but that responsibility for talking about physical activity lies on all health care professionals within the whole health care system. The physiotherapists perceived that they had the most relevant specialist competence among the three professions. Physicians identified that they had the medical responsibility and nurses thought their focus was to work with lifestyle habits. However, all three professions perceived that patients take the physicians’ words a bit more seriously than if the same statement were to come from one of the other professionals. Nonetheless, although the physicians felt responsibility for physical activity, they acknowledged that some physicians may refrain from talking about activity with patients despite a clear need.

PH3: Yes, that is really our responsibility […] the physician first and foremost […] But somehow I think it falls upon all health care professionals who see those patients […]

PH4: I agree with you that it’s our medical responsibility, [we whose opinions] carry the greatest weight in some way, that it falls upon us. After all, it is related to disease. (FGPH2)

#### The own knowledge and competence

Barriers and facilitators related to the participants views on their knowledge and competence to support patients to increased physical activity varied. Some perceived that they had broad competence which also facilitated their work, while others perceived that they had basic competence but thought that it was enough. As a barrier, the physicians perceived a lack of specific knowledge about supporting increased physical activity, resulting in lack of confidence when discussing the matter with patients. For the physiotherapists, a facilitator was their broad knowledge and competence about how to support physical activity. However, a perceived barrier was lack of opportunity to use their broad knowledge and competence, e.g. that it was an uphill job when they found themselves becoming involved in the work only at a very late stage when the patients’ disease had already progressed severely. Another barrier perceived by physiotherapists was a constant need to argue for their expertise, knowledge and competence. The nurses wished for more knowledge about physical activity and perceived, as a barrier, that their workplaces offered less education about preventive work now than earlier in their career. The nurses’ current knowledge had been acquired from further education and from clinical experience in their daily work.

PH4: at some basic level, okay, but when we start getting into specifics I can’t always see a straight path forward in my brain about what I should do first and so on.

PH6: […] I think I’ve got good basic knowledge and that’s enough for almost everyone and it’s fairly easy to give some basic advice. (FGPH1)

#### Collaboration between professions

The participants experienced both barriers and facilitators in the collaboration between different professions.

To work in a team around a patient and refer patients to other professions was perceived as a facilitator since the different professions complement each other and can support the patients in different ways. Collaboration between the professions occurs today but could be both extended and improved. Moreover, the participants wished to have a designated coordinator to streamline the collaboration. As a barrier, lack of collaboration can result in patients receiving different information from different professions, forcing the patient to interpret the information, potentially leading to misunderstandings. The participants had a positive attitude to the professional health educator and saw it as a facilitator. Health educators could complement and unburden other professions, e.g. by working in a more focused manner with lifestyle habits, exercise, PAP, and group sessions. Referring patients to a health educator could work as a link between health care and health promotion. However, a perceived barrier was that it is not yet possible to refer patients to health educators, who are also very few in number at present. The participants also mentioned that it might be hard to assess which patients they should refer to a health educator.

NU3: It’s very important that you have a good collaboration with the physicians […] so the patients don’t get stuck halfway between the physician and the diabetes nurse […] It’s really crucial to be aware that working together as a team is of major importance.

NU2: Yeah, you’re right…

NU1: Mm-hmm (FGNU1)

### The organisation of primary care

This generic category described the participants experiences of how the way in which primary care is organised affects the daily work of supporting patients to increased physical activity. Pertinent factors include support from management, how and how strongly prevention is emphasised, how time and resources are prioritised and what consequences that can have on the participants work, but also how the health care system collaborates with other actors involved in preventive work.

#### Support from management

The participants experienced that the managers’ own interest in the issue of supporting physical activity was reflected in the way management prioritised preventive efforts. This could be either a barrier or a facilitator. For example, at a workplace where the manager was a physiotherapist and had an interest in preventive work, the issue was prioritised, whereas managers who lacked interest might resist efforts to arrange exercise in groups for the patients, since the manager did not identify it as a responsibility of the health care system. To be able to better support the patients’ efforts to increase their physical activity, the participants called for more support from higher level managers within the organisation.

PT1: I also think it’s one of those things, like that we had a manager who was a physiotherapist, so she prioritised it.

PT2: That usually helps.

PT1: Yeah. (FGPT1)

#### Priorities of time and resources, connected to economic consequences

Barriers and facilitators connected to this sub-category included how prioritisation of time and resources affected the daily work of supporting patients to increase physical activity, as well as the economic consequences of prioritisation.

Lack of time and lack of resources (e.g. understaffing in health care professions) were perceived as major barriers for the supportive work. There was seldom enough time and resources to cover the need for support for physical activity, which forced the participants to prioritise between the patients. This resulted in long waiting lists and limited possibility for follow-ups with the patients. Occasionally, the participants admitted to not even raising the issue of physical activity with patients, as time constraints would render the necessary follow-up meetings impossible. However, when the participants perceived that they had time to support the patients, this was seen as a facilitator for the supportive work. Another barrier was the economic compensation system: preventive work was not registered, and was therefore invisible in the statistics on which economic compensation was calculated, resulting in lack of prioritisation of patients in need of support for physical activity. Higher compensation to the clinics would be a major facilitator, and economic consequences of lack of preventive work should be highlighted. A facilitator would be if resources could be prioritised according to the patient load at each health care centre, such that those with highest patient load receive more resources; this could contribute to more equitable care, e.g. in socioeconomically disadvantaged areas. Continuing to support patients with metabolic risk to increase their physical activity is a complex task, since the number of patients is constantly increasing, at the same time as the resources available within the health care organisation are becoming ever more scanty.

NU1: We have quite a lot of patients, diabetes patients, we have 600 out of 8000 [patients] registered at my health care centre.

NU2: WOW!

NU1: And there are two of us, diabetes specialist nurses, and neither of us working full-time with diabetes […] We don’t have time, that I can tell you! […] we don’t dedicate as much time to that as I wish we could. (FGNU1)

#### The role of prevention and routines within primary care

Barriers and facilitators within this sub-category were connected to which routines were established at the workplaces, the participants view on preventive work within health care, and how preventive work could be organised.

Routines for preventive work existed at some workplaces, acting as a facilitator, while routines were lacking at others, which was perceived as a barrier. Routines could involve various types of appointments, or medical record templates, or to report to the National Diabetes Register. The routines affected prioritisations of how much time the participants could give to the patients. Also, some workplaces set goals for their preventive work, which was perceived as a facilitator since it generated regular discussion and follow-up within the workplace. The participants agreed on the importance of working with prevention within the health care system. On the one hand, as a facilitator, they perceived that primary care centres are doing their best and there is great interest in supporting patients to physical activity. On the other hand, perceived as a barrier, preventive work should have higher priority within primary care, as primary care should be a role model. Also, preventive work within the health care system could be better organised; large differences exist today between different health care centres, which therefore frequently ‘reinvent the wheel’ instead of collaborating. Another barrier was the lack of clarity concerning who owns the responsibility for prevention, what is the patient’s own responsibility, what is the responsibility of health promotion, what is the health care system responsible for, and where can society add resources to work more with prevention.

PH5: It’s really a part of the treatment, both preventing and treating […] On the other hand, in general I think that those of us who work in health care should help those who need support […]

PH6: Yeah, well I think just the same thing, that it’s here within primary care that we should sort of set a good example and really latch onto them [patients at risk] and work with prevention.” (FGPH1)

#### Primary care collaboration with other actors

Barriers and facilitators within this sub-category were related to how different actors in society collaborate with primary care and the consequences this may have for patients.

To refer patients from health care professionals to actors in society was perceived as a barrier, since there was no system for such referral. The participants wished for more possibilities to refer patients, e.g. collaboration with sports clubs or other organisations. Another barrier was that whereas organised physical activity within the healthcare system was subsidised, physical activity in the wellness sector was not, a reality that patients disliked. However, the participants also perceived that referring patients to other actors could be a facilitator since it could contribute to more equitable care if patients from different socioeconomic backgrounds could be referred to the same place. Prevention was seen as a method to counterbalance socioeconomic gaps. Moreover, the patient’s socioeconomic position was perceived to affect the patient’s physical activity: those with lower socioeconomic position were less physically active than those with higher socioeconomic position.

PT1: When they leave us [the health care system] it’s get back out and stand on you own two legs again. Maybe with support from others, other opportunities that exist in society.

PT2: It feels like we’re with them at the start, let’s say 2-3 meetings depending on which patient you have in front of you. But later, once they’ve got started, you really want them to do some physical exercise whether it’s at a gym or some aerobics group or something so they can do it on their own further on. (FGPT1)

## Discussion

The aim of this study was to explore how health care professionals within Swedish primary care experience barriers and facilitators in their work to support persons with metabolic risk factors to increase their physical activity.

The study found that health care professionals perceived barriers and facilitators for supporting people with metabolic risk factors at several levels that should, according to implementation research [22], be considered when working to improve the support. The levels of barriers and facilitators in the current study included personal factors, such as the patient’s attitude to physical activity and the health care professionals’ own views about support work, their knowledge, and their collaboration with other professions and actors. Another level included organisational factors, such as lack of time, economic constraints, and support from management.

### Individual factors

The result showed that the participants agreed on the importance of supporting patients to increase their physical activity, and that it felt motivating if they managed to give the patients the support they required. A systematic review based on studies from several countries as well as different health care settings (primary care included) found similar results, reporting that health care professionals agreed about the importance of physical activity for their patients and thought that physical activity promotion was part of their professional role [20]. Other studies within Swedish primary care found that health care professionals express a willingness and a desire to work more with prevention [29,30]. It has been found that health care professionals’ enthusiasm for physical activity promotion was associated with such factors as receiving training in physical activity promotion, perceiving self-efficacy for promotion, assessing patients’ physical activity levels, and belief in the benefits of physical activity [31]. It has also been shown that Swedish and American patients want support with lifestyle counselling from primary care [21].

Individualising the support was a facilitating factor, and was described by the participants as involving more in-depth conversations with the patients, often based on motivational interviewing. Joelsson et al. evaluated experiences of using physical activity on prescription (PAP) for patients with metabolic risk factors with low physical activity levels within the Swedish primary care context [32]. They concluded that tailoring the physical activity support, as well as having regular follow-ups, can contribute to increased and maintained physical activity and motivation among the patients [32].

The current study showed that professionals’ own knowledge and competence about supporting increased physical activity varied between the professions. Similarly, a study by Kardakis et al. found differences between professions; however, they state that the professionals in general reported themselves as being thoroughly knowledgeable [30]. In contrast, a systematic review showed that health care professionals perceived that they had some knowledge about physical activity promotion but needed more training [20].

Collaboration between professions and working in teams when interacting with the patient was perceived as a facilitator in this study. Collaboration with actors outside the health care context was something the participants wished for, but it was currently perceived as a barrier since there was no system for such collaborations. Kettle et al. found that support delivered by primary care professionals together with other actors significantly increased physical activity compared to when it was delivered by the health care sector alone [19]. The current study also addressed health educators as a complementary profession, which the participants viewed as a facilitator. However, systems for referring patients to health educators are lacking within the Swedish health care system today. The systematic review by Albert et al. found that to broaden the treatment of chronic diseases, explicit strategies are required, including preventive and integrative physical activity promotion approaches [20]. These strategies are needed both between the health care professionals working within health care, but also between them and physical activity specialists working outside the system. Moreover, referral pathways need to be evaluated and improved [20].

In summary, at an individual level, the participants perceived facilitators related to a willingness to support the patients to increased physical activity; they saw the importance of prevention, of individualising the support, and of collaborating with other professions. However, the perceived competence in supporting physical activity varied between the professions.

### Organisational factors

A barrier perceived by the participants was that preventive work does not result in economic compensation since it was not registered in the statistics; this contributed to low prioritisation of prevention efforts. The results also showed that the amount of supportive work done with the aim of increasing physical activity did not reach the desired level; important barriers in this context included lack of time and resources. This also has been concluded by other studies [20,29,33–35] and confirmed by a national report on preventive work in Sweden [12,36,37]. The participants highlighted that they sometimes actively decide not even to raise the issue of physical activity with patients, as time constraints would make the necessary follow-up meetings impossible. Follow-up has been shown to be a key factor to achieve behaviour change and increase physical activity within primary care [19]. The lack of time also forced the professionals to prioritise between patients.

Another barrier found in this study was the lack of clarity concerning who is responsible for prevention: is it the patient, the health care system, health promotion, or society as a whole? Persson et al. found in a focus group discussion study that general practitioners perceived increasing physical activity as a shared responsibility for society, the care team and the patient [38]. Moreover, all three types of professionals included in this study (nurses, physicians, and physiotherapists) thought that their own profession should be responsible for supporting physical activity. Other studies have found similar opinions among nurses [39] and physiotherapists [35].

The current study found that management can work either as a barrier or a facilitator for the participants to be able to support the patients. Studies within Swedish primary care identified several preconditions required to enable implementation of physical activity on prescription in Swedish primary care, including support at different levels of management, and from local and central support structures [39,40]. Kardakis et al. found that management at health care centres was supportive and had a positive impact on health promotion. However, structures to sustain the health promotive work were scarce [30].

In summary, at an organisational level, the participants perceived barriers related to lack of time and resources, and unclarity about the responsibility for prevention; management could be perceived as both a barrier and a facilitator.

### The complexity of supporting physical activity

Many of the factors highlighted as facilitators in this study, and confirmed by other studies, were at the personal level and were time consuming. In addition, factors highlighted as barriers at an organisational level included lack of time and resources. Together, these observations indicate that support for physical activity is a complex issue that needs to be handled at an organisational level rather than being seen as a responsibility for each health care professional.

According to the implementation framework CFIR, an intervention (in this case, to improve the support for people with metabolic risk factors to increase their physical activity) should be adapted to the setting where it occurs by including all persons involved in the intervention (both health care professionals and managers). The inner setting includes the cultural, structural, and political aspects, and the outer setting includes the social, political, and economic context where the implementation will occur, in this case, a primary care centre. The interplay between the individual professionals and the organisation where they work is important to consider. The individuals involved in the implementation are of importance since they have an impact through their individual, professional, organisational, and cultural mindset, but also their interests, norms and affiliations. The implementation process can comprise several subprocesses which can occur simultaneously at multiple levels in the organisation [22]. Implementation research, together with the results from this study, can be helpful for health care decision-makers attempting to improve the preventive work regarding support to increased physical activity within primary care.

### Strengths and limitations

Focus groups were chosen because it is a method where the moderator acts only as a moderator and the data are generated from high-quality dialogue between the participants [25]. This study took place during the vaccination phase of the Covid-19 pandemic when Sweden’s primary health care system was under severe pressure, which made the recruitment challenging. The intention was to recruit participants working in areas with different socioeconomic prerequisites and participants with both positive and negative attitudes towards supporting physical activity; however, this was not feasible. Also, a more equal gender distribution was desired. The resulting homogeneity of the participants can affect the transferability of the results [41]. One of the focus groups included only two participants due to the recruitment challenge. This could have affected the richness and depth in the data. However, since the interviews were conducted digitally, it was possible to include participants from five different regions in Sweden. Also, the participants included in the study all had experience of supporting people with metabolic risk factors within the primary care, which is important for the credibility. The transcripts of the focus group discussions were not returned to the participants for comments, a procedure that could have strengthened the credibility. The analysis was conducted with a manifest approach which is close to the text and provides clear categories [42]. This approach was chosen as it was deemed to be the best fit for the aim of the study, and also to make the results usable for health care professional within the clinical setting. KL was doing qualitative content analysis for the first time, but close collaboration with the experienced ÅN, involving continuous discussion of codes and selection of supporting quotes for each sub-category, ensured dependability of the results. Credibility was ensured by KL and ÅN independently selecting meaning units and then matching them. The participants did not receive feedback on the findings. This can have an impact on the dependability of the results since feedback could elicit corrections from the participants if something had been misunderstood.

## Conclusions

The findings from this study suggest that barriers and facilitators for supporting physical activity among patients with metabolic risk factors can be found at several levels within primary care, from individual patients and health care professionals to the organisational level. At all levels, responsibility, prioritisation, and the complexity of behaviour change regarding physical activity were highlighted, as well as time and resources as important prerequisites for supporting patients’ efforts to increase their physical activity. In the primary care setting, these aspects should be addressed when implementing support to increase physical activity in people with metabolic risk factors.

## Appendix A.

### The COREQ checklist

Developed from:

Tong A, Sainsbury P, Craig J. Consolidated criteria for reporting qualitative research (COREQ): a 32-item checklist for interviews and focus groups. Int J Qual Health Care. 2007;19(6):349–357.

**Table:**

No. item	Description	Section #
Domain 1: Research team and reflexivity		
Personal characteristics		
1. Inter viewer/facilitator	Which author/s conducted the interview or focus group?	Data collection
2. Credentials	What were the researcher’s credentials? E.g. PhD, MD	Data collection
3. Occupation	What was their occupation at the time of the study?	Data collection
4. Gender	Was the researcher male or female?	Data collection
5. Experience and training	What experience or training did the researcher have?	Data collection
Relationship with participants		
6. Relationship established	Was a relationship established prior to study commencement?	Data collection
7. Participant knowledge of the interviewer	What did the participants know about the researcher? e.g. personal goals, reasons for doing the research	Data collection
8. Interviewer characteristics	What characteristics were reported about the inter viewer/facilitator? e.g. Bias, assumptions, reasons and interests in the research topic	Data collection
Domain 2: Study design		
Theoretical framework		
9. Methodological orientation and Theory	What methodological orientation was stated to underpin the study? e.g. grounded theory, discourse analysis, ethnography, phenomenology, content analysis	Data analysis
Participant selection		
10. Sampling	How were participants selected? e.g. purposive, convenience, consecutive, snowball	Setting and participants
11. Method of approach	How were participants approached? e.g. face-to-face, telephone, mail, email	Setting and participants
12. Sample size	How many participants were in the study?	Setting and participants
13. Non-participation	How many people refused to participate or dropped out? Reasons?	Setting and participants
Setting		
14. Setting of data collection	Where was the data collected? e.g. home, clinic, workplace	Data collection
15. Presence of non-participants	Was anyone else present besides the participants and researchers?	Data collection
16. Description of sample	What are the important characteristics of the sample? e.g. demographic data, date	Data collection
Data collection		
17. Interview guide	Were questions, prompts, guides provided by the authors? Was it pilot tested?	Data collection
18. Repeat interviews	Were repeat inter views carried out? If yes, how many?	Data collection
19. Audio/visual recording	Did the research use audio or visual recording to collect the data?	Data collection
20. Field notes	Were field notes made during and/or after the inter view or focus group?	Data collection
21. Duration	What was the duration of the inter views or focus group?	Data collection
22. Data saturation	Was data saturation discussed?	We took a pragmatic approach and decided that it was enough, as the tendency was that no new information emerged.
23. Transcripts returned	Were transcripts returned to participants for comment and/or correction?	Discussion, strengths and limitations
Domain 3: Analysis and findings		
Data analysis		
24. Number of data coders	How many data coders coded the data?	Data analysis
25. Description of the coding tree	Did authors provide a description of the coding tree?	Data analysis
26. Derivation of themes	Were themes identified in advance or derived from the data?	Data analysis
27. Software	What software, if applicable, was used to manage the data?	Data analysis
28. Participant checking	Did participants provide feedback on the findings?	Discussion, strengths and limitations
Reporting		
29. Quotations presented	Were participant quotations presented to illustrate the themes/findings? Was each quotation identified? e.g. participant number	Results
30. Data and findings consistent	Was there consistency between the data presented and the findings?	Results
31. Clarity of major themes	Were major themes clearly presented in the findings?	Results
32. Clarity of minor themes	Is there a description of diverse cases or discussion of minor themes?	Results

## Appendix B. The Topic guide

### Introductory question

- What is your general opinion about supporting patients to increased physical activity?

### Domain: description of the meeting with the patient

General instructions: Think about meetings with your patients where you have tried to support the patients to increase their physical activity.

Questions:

- How did you raise the issue?
- How did you identify patients in need of support for physical activity?
- Can you describe the meetings in terms of what you perceive worked well?
- Can you describe if there was anything that you perceive did not work so well?
- Can you describe if there was something that you wanted to do differently?

### Domain: health care professionals’ views of the patients’ need for physical activity support

Questions:

- Can you describe how the patients usually react when you ask them about their physical activity?
- Do you perceive that you can give the patients the support they ask for?
- Do you use any tools to support the patients to increase their physical activity (e.g., physical activity on prescription)?

### Domain: health care professionals’ own knowledge and competence

Questions:

- Can you describe how you view your own knowledge and competence about supporting physical activity?
- What opinion concerning whether any specific health care profession should have greater responsibility for work to support patients to physical activity?
- Are you familiar with the profession health educator? (If the answer is no, tell them about the profession.)
- Do you think that health educators could be useful in the work to support patients to increase their physical activity?
- What is your opinion about referring patients whom you consider need support to other professions or actors?

### Domain: the workplace and the organization

Questions:

- If you think about your workplace and your colleagues, what is the general opinion about supporting patients to increase their physical activity?
- Does your workplace have clear routines for supporting patients to increase their physical activity?
- If yes, can you tell us a bit more?
- If no, what would you need in terms of routines to facilitate the work with supporting patients to increase their physical activity?
- Does your workplace have clear goals for supporting patients to increase their physical activity?
- If yes, can you tell us a bit more?
- If no, what would you need in terms of goals to facilitate the work with supporting patients to increase their physical activity?
- Can you describe how your manager reasons about the work with support patients to increase their physical activity?
- What would you need from your manager to facilitate the work with supporting patients to increase their physical activity?
- Can you describe whether you work together with people from other professions at your workplace to support patients to increase their physical activity?

### Closing questions

- Of all the things we have discussed today, what do you think was most important?
- Did we miss anything relevant in the discussions that we had today? Is there anything more that you want to discuss?

## Appendix C. The Overview of generic categories and sub-categories, and barriers and facilitators experienced by the health care professionals

**Table:**

Generic categories	Patient readiness for change	Supporting the process of change	The professional role	The organisation of primary care
*Sub-categories*	*The patients’ attitude to physical activity*B: Low knowledge and self-awareness F: High knowledge and self-awareness	*The conversation that promotes motivation and behavior change*B: Lack of patient motivation F: Creating a positive climate of change F: Individualise to the patient	*The health care professional’s own view* B: Hard to support patients with MetS risk factors F: The importance of the supportive work F: Manage to give the patients’ the support they require	*Support from management*B: Mangers not interested in supporting physical activity F: Mangers are interested in supporting physical activity
	*The patients’ insight to their own disease*B: Feeling guilt and shame B: Patients with no established type 2 diabetes F: Patients with established type 2 diabetes	*Usability of tools for supporting physical activity*B: Hard to use physical activity on prescription F: Several practical methods	*The view of the profession responsible for physical activity*B: Physicians may disregard from talking about physical activity F: All professions perceived that they were responsible	*Priorities of time and resources connected to economic consequences*B: Lack of time and resources B: The economic compensation F: Enough time and resources F: More economic compensation
			*The own knowledge and competence*B: Lack of tools to support B: Lack of knowledge F: Having enough tools to support F: Having enough knowledge	*The role of prevention and routines within primary care*B: Lack of routines for the preventive work B: Low prioritization for preventive work B: Lack of collaboration between health care centers F: Having routines for the preventive work F: Having goal setting for the preventive work F: Large interest for prevention
			*Collaboration between professions*B: Lack of teamwork F: Work in team F: Refer patients to other professions F: Health educators as complement	*Primary care collaboration with other actors*B: Lack of system for referring patients to other actors B: Different prices for physical activity at different actors F: Referring patients to other actors F: Early prevention in school

B: Barriers; F: Facilitators; MetS: Metabolic syndrome.
